# Supplementary figures and images for: cnm-positive Streptococcus mutans is associated with galactose-deficient IgA in patients with IgA nephropathy
Source: PLoS One. 2023 Mar 2;18(3):e0282367. doi: 10.1371/journal.pone.0282367 (PMC9980772; doi:10.1371/journal.pone.0282367)

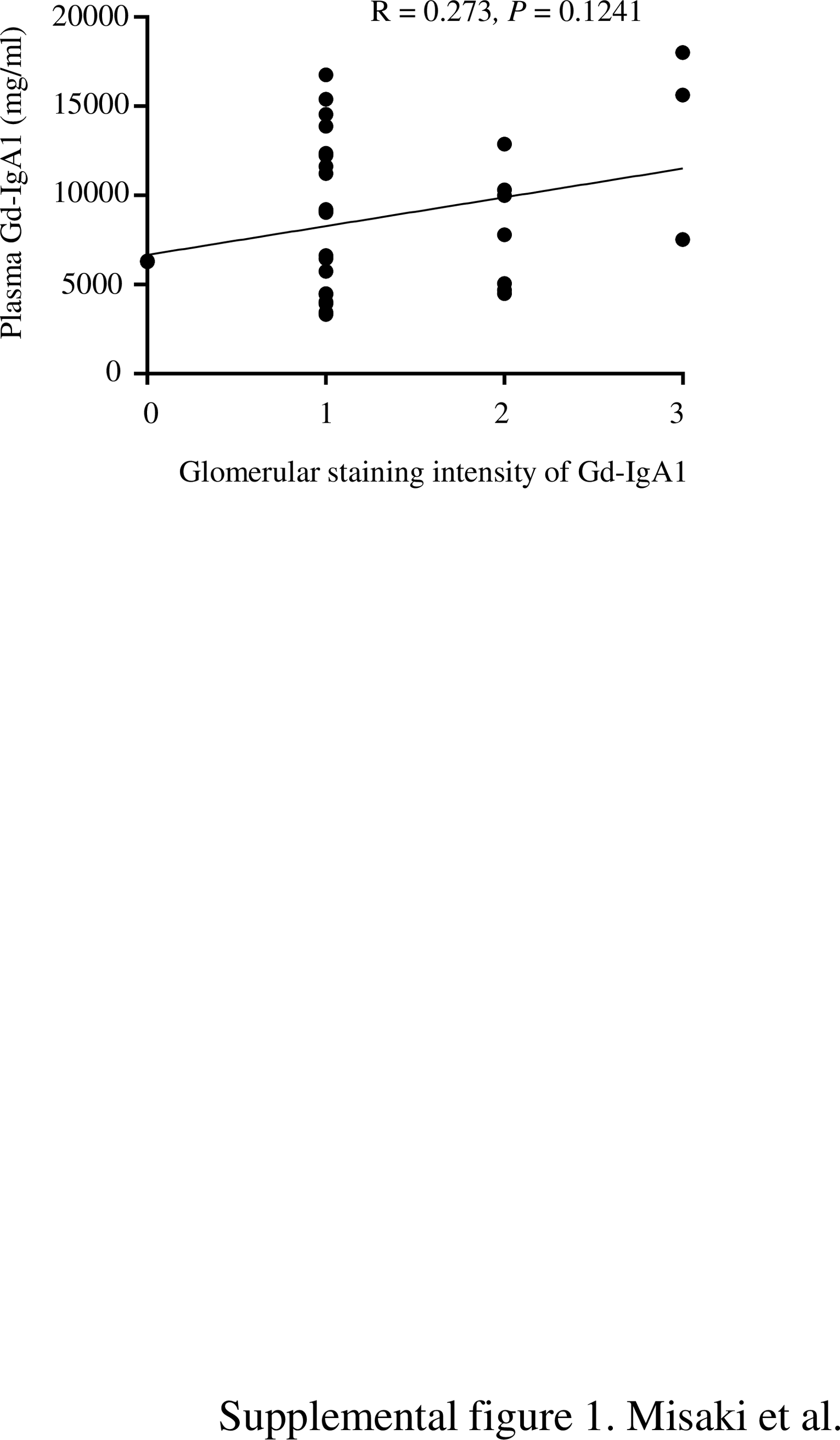

Supplement: S1 Fig — There was no significant association between the glomerular staining intensity of Gd-IgA1 and the plasma Gd-IgA1 concentration. The data were examined for statistical significance using a simple regression analysis. Gd-IgA1: galactose-deficient IgA1. (TIF) [file pone.0282367.s001.tif]

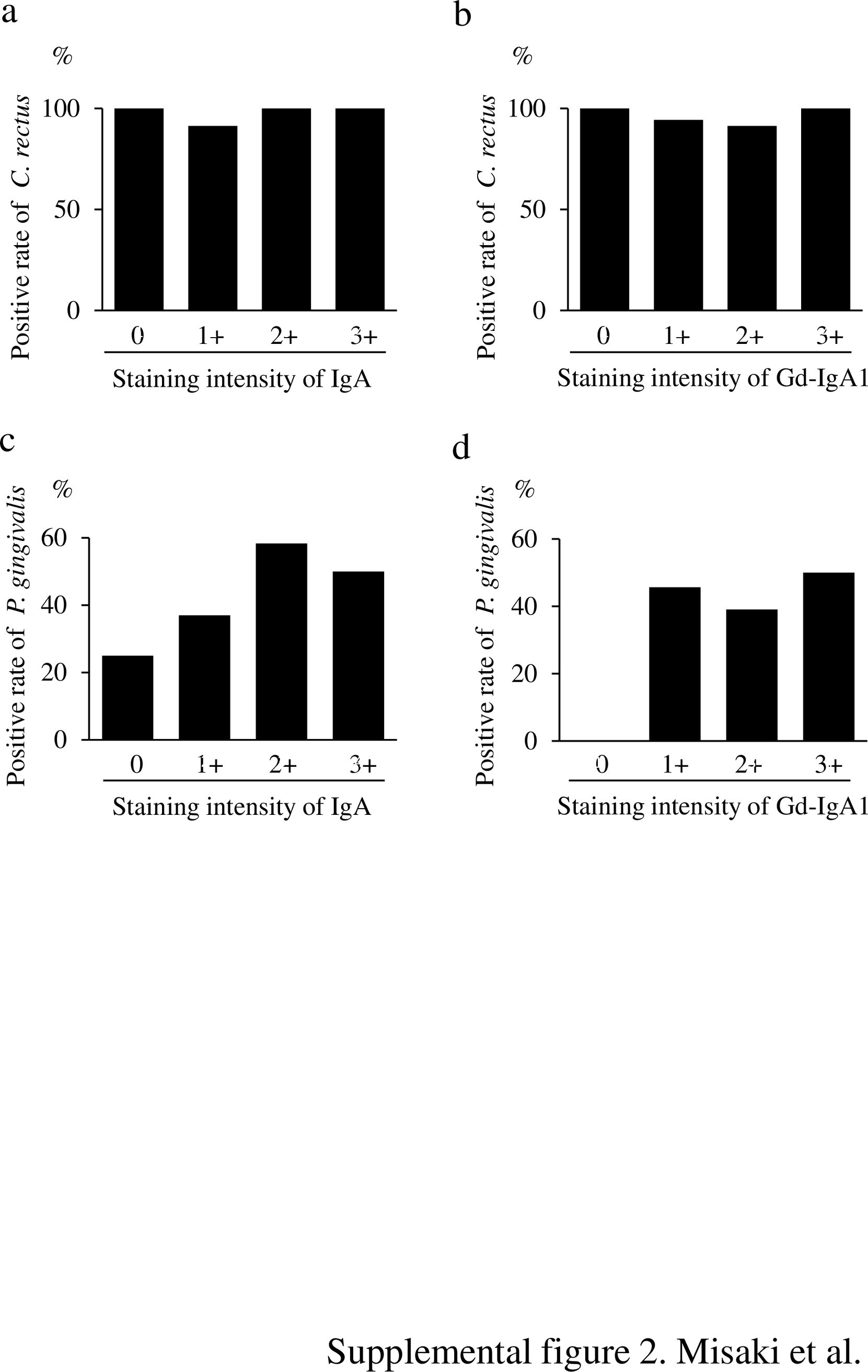

Supplement: S2 Fig — Comparison of the glomerular staining intensity of IgA and the positive rate of Campylobacter rectus in the oral cavity (a). Comparison of the glomerular staining intensity of Gd-IgA1 (KM55) and the positive rate of C. rectus in the oral cavity (b). Comparison of the glomerular staining intensity of IgA and the positive rate of Porphyromonas gingivalis in the oral cavity (c). Comparison of the glomerular staining intensity of Gd-IgA1 (KM55) and the positive rate of P. gingivalis in the oral cavity (d). The data were examined for statistical significance using the Cochran–Armitage trend test. P < 0.05 was considered statistically significant. (TIF) [file pone.0282367.s002.tif]
